# Transcriptomic Analysis of Marine Gastropod *Hemifusus tuba* Provides Novel Insights into Conotoxin Genes

**DOI:** 10.3390/md17080466

**Published:** 2019-08-10

**Authors:** Ronghua Li, Michaël Bekaert, Luning Wu, Changkao Mu, Weiwei Song, Herve Migaud, Chunlin Wang

**Affiliations:** 1 Key Laboratory of Applied Marine Biotechnology, Ministry of Education, Ningbo University, Ningbo 315211, China; 2 Institute of Aquaculture, Faculty of Natural Sciences, University of Stirling, Stirling FK9 4LA, Scotland, UK; 3 Collaborative Innovation Center for Zhejiang Marine High-efficiency and Healthy Aquaculture, Ningbo University, Ningbo 315211, China

**Keywords:** *Hemifusus tuba*, transcriptome, conotoxin, simple sequence repeats

## Abstract

The marine gastropod *Hemifusus tuba* is served as a luxury food in Asian countries and used in traditional Chinese medicine to treat lumbago and deafness. The lack of genomic data on *H. tuba* is a barrier to aquaculture development and functional characteristics of potential bioactive molecules are poorly understood. In the present study, we used high-throughput sequencing technologies to generate the first transcriptomic database of *H. tuba*. A total of 41 unique conopeptides were retrieved from 44 unigenes, containing 6-cysteine frameworks belonging to four superfamilies. Duplication of mature regions and alternative splicing were also found in some of the conopeptides, and the de novo assembly identified a total of 76,306 transcripts with an average length of 824.6 nt, of which including 75,620 (99.1%) were annotated. In addition, simple sequence repeats (SSRs) detection identified 14,000 unigenes containing 20,735 SSRs, among which, 23 polymorphic SSRs were screened. Thirteen of these markers could be amplified in *Hemifusus ternatanus* and seven in *Rapana venosa*. This study provides reports of conopeptide genes in Buccinidae for the first time as well as genomic resources for further drug development, gene discovery and population resource studies of this species.

## 1. Introduction

Marine organisms represent half of the total global biodiversity, and as such, they provide an abundance of chemical space to be explored for peptide-based drug discovery [1]. The toxic peptides found in marine organisms, such as jellyfish, cone snails, sea urchins and lionfish, are of great biotechnological interest for applications in medicine. Possible use could be as biochemical tools in neurophysiological studies and for the discovery of new molecular targets in pharmacology [2]. A diverse range of predatory marine gastropods produce toxins (e.g., Conoidae superfamily), yet most of these molecules remain unknown or uncharacterised [3].

*Hemifusus tuba* (Gmelin, 1791), the tuba false fusus, is a marine gastropod of the family Melongenidae. Its natural range extends from the Sea of Japan through the South China Sea to the Philippines [4]. *H. tuba* is served as luxury food in some Asian countries, popular for its delicious taste and high nutritive value. The economic value of *H. tuba* has led to increased research efforts in population genetics [5], conservation [6,7] and aquaculture studies [8,9]. While there is some knowledge on the species biology, including the circulatory and respiratory systems [4], reproduction and behaviour [10,11,12,13] and physical characteristics (nutritional composition and microstructure of the conch shell) [9,14], there is a complete lack of molecular understanding for bioactive compounds discovery and for the population resource management of this species.

Conotoxins are conopeptides with generally 12−35 amino acids. Initially described in the cone snail, they have remarkable molecular diversity and have the potential to target neuroreceptors, ion channels and transporters, with high potency and specificity. They can be bioactive but not specifically toxic. These attributes make it an attractive candidate for the treatment of neuropathic pain and acute pain [15]. While *Conus* species have been in the spotlight of drug discovery, several other marine gastropod species have been reported to contain bioactive compounds with potential bioactivity, but most of these compounds have not yet been identified and characterised [3].

Whelks have been used in traditional Chinese medicine for hundreds of years; they were first mentioned in “Bencao Shiyi”, by Chen Cangqi, around 739 A.D. “Medicinal Fauna of China” [16] records *H. tuba* as medicine to treat lumbago and deafness. However, its active ingredients and molecular mechanisms have not been characterised. The recorded therapeutic effect of *H. tuba* for pain relief suggests that it may contain chemical compounds somewhat similar to *Conus* species.

With the development of high-throughput sequencing platforms, the transcriptome of many economically important, non-model species of molluscs have been extensively investigated [17], leading to the discovery of new bioactive compounds, physiological pathways and insights into the mechanisms of evolution [18,19,20,21]. High-throughput sequencing methods have also shown great potential in screening for polymorphic simple sequence repeats (SSRs or microsatellites) used as tools for species conservation and sustainable aquaculture production such as studies on population diversity, conservation genetics, marker-assisted selective breeding and evolutionary analyses [17,22].

The purpose of this study was to develop transcriptomic resources using High-throughput sequencing to facilitate functional transcript discovery and population genetic study in *H. tuba* species. Such new transcriptomic information supports bioactive compound discovery, phylogenetics and population genetic studies for the exploitation of this commercially important species in the future.

## 2. Results

### 2.1. High-Throughput Sequencing and De Novo Assembly

The Illumina sequencing of *H. tuba* visceral mass tissue generated 33,546,714 raw paired-end reads. The reads were deposited in the European Bioinformatics Institute (EBI) European Nucleotide Archive (ENA) project ID PRJEB30840. A total of 22,892,498 paired-end reads (68.2%) passed the pre-processing filters, and a final 21,397,329 (63.8%) passed the mRNA cleaning step and were used during the de novo assembly process (Table 1).

**Figure 1:**
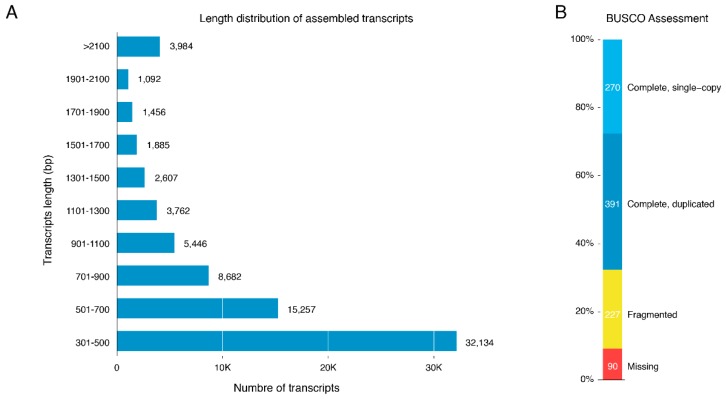
*H. tuba* transcript assessments. (**A**) Length distribution of the assembled *H. tuba* transcript. Clean reads for *H. tuba* were assembled and resulted in 76,306 transcripts. (**B**) BUSCO assessment (Metazoa database; number of BUSCO, 978).

The final assembly reconstructed a total of 76,306 transcripts with an average size length of 824.6 nt and an N_50_ length of 1014.0 nt (Table 1 and Figure 1A). The high-quality (cleaned) reads were then mapped back to the assembled transcripts to assess the quality of the assembly; as a result, 54.5% of the reads were successfully mapped to the assembled final transcriptome, while 82.9% were mapped to the raw/unfiltered transcriptome. A Benchmarking Universal Single-Copy Orthologs (BUSCO) completeness assessment recovered 90.8% of near-universal single-copy orthologs selected from the Metazoa database (Figure 1B). The clustering of the transcripts generated 61,575 unigenes (a set of transcripts/isoforms that stem from the same transcription locus, i.e., gene) with a mean length of 744.2 nt and an N_50_ length of 865.0 nt (Table 1). The assembly was deposited at the European Bioinformatics Institute (EBI), analysis ID ERZ976199.

### 2.2. Annotation and Functional Characterisation of the H. tuba Transcriptome

**Figure 2:**
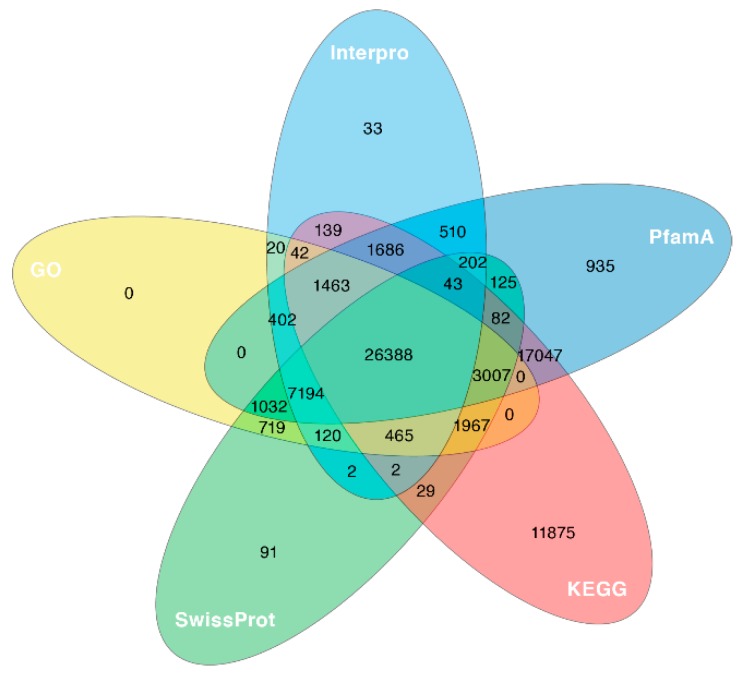
A five-way Venn diagram. The figure shows the unique and overlapped transcripts showing predicted protein sequence similarity with one or more databases (details in Table 2).

The predicted proteins from the reconstructed transcripts were subjected to BlastP similarity searches against SwissProt, Pfam, InterPro, Kyoto Encyclopedia of Genes and Genomes (KEGG) and Gene Ontology (GO) databases. Of the total of 76,306 transcripts, 75,620 (99.1%) were annotated by at least one database, and 26,388 (34.6%) were annotated in all five databases used (Table 2 and Figure 2).

**Figure 3:**
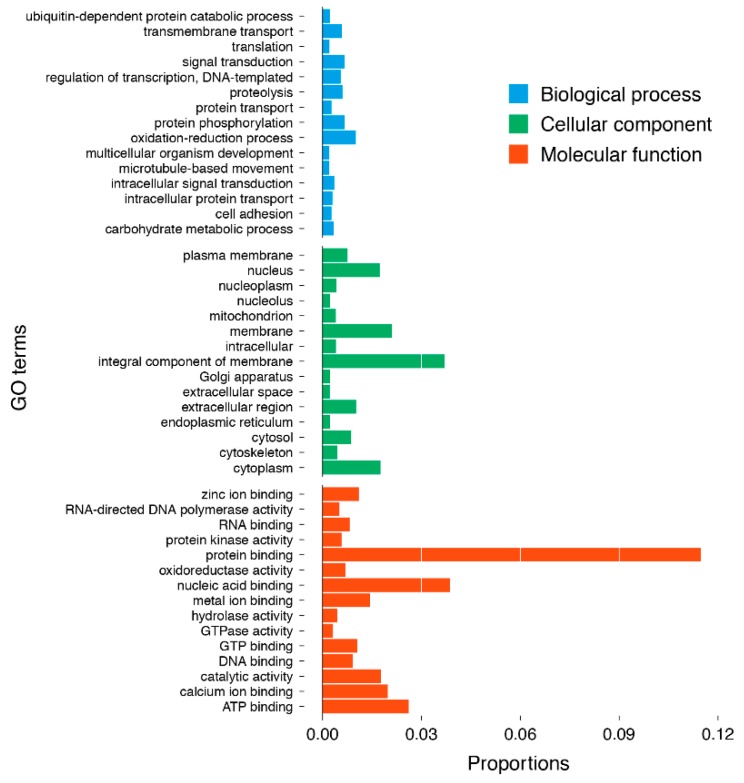
Level 2 GO annotations using the gene ontology (GO) of assembled transcripts.

A total of 42,819 assembled transcripts were allocated into three major GO classes: 11,570 (27.02%) transcripts were allocated into “biological processes”; 10,268 transcripts (23.98%) into “cellular components”; and 20,981 (49.00%) into “molecular functions” (Figure 3). In the biological process category, the oxidation–reduction process (432 transcripts), protein phosphorylation (287 transcripts) and signal transduction (281 transcripts) were the most abundant groups. In cellular component terms, the integral component of membrane (1583 transcripts), membrane (903 transcripts), cytoplasm (752 transcripts) and nucleus (743 transcripts) were the dominant groups. Under the molecular function category, protein binding (4915 transcripts), nucleic acid binding (1656 transcripts) and ATP binding (1119 transcripts) were the most abundant groups.

### 2.3. Conopeptide Identification

Literature reported the conotoxin protein/transcript/gene as a “precursor”, while the mature toxin peptide region is referred as a “conopeptide”. This leads to confusion when one precursor contains more than one toxin peptide region/conopeptide domain. For clarity, we are using “conotoxin protein” and “conopeptide”.

**Figure 4:**
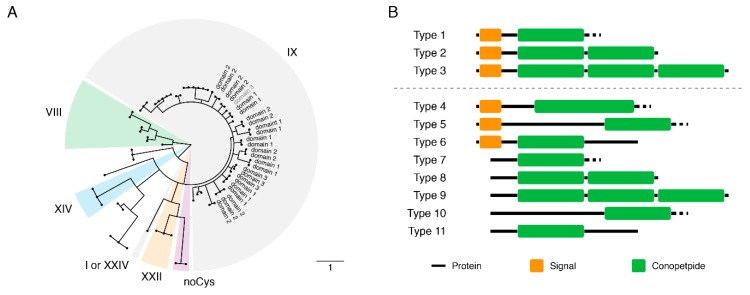
Conopeptides summary. (**A**) Conopeptide tree based on the alignment of the 73 peptides classified. The Cys framework is reported on the outer section of the tree. Every conopeptide in a multi-domain protein was included in the analysis. Domains aligned with respective domain of duplicated genes. (**B**) Structure of conotoxin proteins. Types 1, 2 and 3 were complete transcripts, while 4 to 11 were truncated transcripts or incompletely characterised proteins.

In order to capture the diversity of conotoxin, the predicted protein translation of all transcripts was used to conduct an initial BlastP search against the known conopeptide sequences. A total of 66 transcripts were identified as coding for putative conotoxin proteins (Supplementary Table S1). A total of 82 conopeptide domains (toxin peptide region; Supplementary Table S2) were extracted from the initial search. Following further classification using a conservative BlastP (*e*-value threshold < 10^−10^) and both ConoSorter and ConoDictor analysis, a total of 58 transcripts (from 45 unigenes) were categorised as coding conotoxin proteins and 73 conopeptide domains were clustered (Figure 4A).

As these peptides were small and very polymorphic, the gene tree generated was of low discrimination. After removing these identical/high similarity sequences, 41 unique conopeptides were retrieved as described below:

1) The structure of most conotoxin proteins identified in this study generally consists of three distinct regions: a N-terminal signal peptide region, a less-conserved intervening pro-peptide region (pro-region), and a hypervariable C-terminal mature toxin region (conopeptide). Of the 58 distinct conotoxin transcripts identified, 31 contain the complete coding sequences (CDS) including the signal-pro-mature toxin canonical structure and 27 were partial and incomplete (Figure 4B).

2) Of the 41 unique conopeptides, 40 were disulphide-rich conopeptides, which contained two or more disulphides, and a total of six patterns of Cys frameworks, the most abundant one being the type “IX” with 6-Cys residues arranged in the pattern “C-C-C-C-C-C” (Table 3).

3) According to the BlastP results of the signal peptide region, four super-families homologous to the peptide of *Conus* species are reported (O1, O4, Divergent MTFLLLLVSV, and Divergent MKVAVVLLVS), where the most abundant one was the “Divergent MTFLLLLVSV”.

4) Eleven conotoxin proteins contained multiple conopeptides (up to three; Figure 4B). There were four proteins encoding three conopeptides and seven encoding two conopeptides. The multiple conopeptides, from the same protein, were different from each other.

5) The conopeptide domains of multiple-domain proteins were more similar between proteins than between domains. This suggests old domain duplications, followed by more recent gene duplications. Gene duplication was detected in nine of the 58 distinct conotoxin protein sequences.

6) To investigate the relative transcription levels of different conotoxin proteins, we report the abundance of each conotoxin transcript (expressed in number of fragments per kilobase of transcript per million mapped fragments, FPKM; Supplementary Table S1).

### 2.4. Characterisation and Validation of Microsatellites

A total of 20,735 SSR loci were detected in 14,000 (22.7%) sequences out of 61,575 unigenes, including 6975 di-nucleotide, 11,654 tri-nucleotide, 1812 tetra-nucleotide, 278 penta-nucleotide and 16 hexa-nucleotide type repeats (Table 4). The frequencies of AC/GT and AG/CT were highest in di-nucleotide repeats, accounting for 61.1% and 35.3% of di-nucleotide SSRs, respectively. AT and TA repeats accounted for 1.6% and 0.9% of di-nucleotide SSRs respectively. Within the tri-nucleotide repeats, AGC/CTG (22.6%), followed by ACC/GGT (21.7%) were the most repeated motifs.

Fifty-eight microsatellite-containing sequences were selected for microsatellite marker optimisation and validation because of their repetition times and flaking sequence priority. Of the 58 primer pairs, 22 were not amplified, 13 produced monomorphic profiles and 23 were polymorphic among 30 individuals of *H. tuba*. The characteristics of these polymorphic loci are shown in Supplementary Table S3. The number of alleles ranged from 2 to 9. The observed and expected heterozygosity ranged from 0.23 to 1.00 and 0.24 to 0.87 with an average of 0.85 and 0.68, respectively. The ranges of polymorphic information content (PIC) were from 0.22 to 0.84. Nine of the 23 loci showed significant departure from the Hardy–Weinberg equilibrium after a sequential Bonferroni correction and no significant pairwise linkage disequilibrium between any loci (*p*-value < 0.001).

All 23 polymorphic SSR loci were subsequently used in cross-species amplification tests in two other related species. Thirteen of the 23 markers could be transferred to *H. ternatanus* and 7 could be amplified in *R. venosa* (Table 5).

## 3. Discussion

*H. tuba* is a valuable species for food and potential marine resources discovery; however, bioactive compound exploitation and fisheries’ management are hindered by the lack of genomic resources available. This study presents the first transcriptome analysis of the marine whelk *H. tuba*. Key findings include the identification of conopeptides from a member of the Buccinidae family, which may lead to new bioactive compounds and a number of microsatellite markers that can be used for population genetic studies and resource management programmes.

The average length of the unigenes obtained (744.2 nt) was comparable to recently published transcriptomes in other gastropod species such as *R. venosa* (619.0 nt; [23]), *Pomacea maculata* (878.0 nt; [24]), *Koreanohadra kurodana* (678 nt; [25]) and *Clithon retropictus* (736.9 nt; [22]). The transcriptome completeness of *H. tuba* was assessed using BUSCO and showed 90.8% of ortholog genes present, which confirmed the good coverage of the total gene content of this species and the overall robustness of the transcriptome sequencing, assembly and annotation pipeline.

Peptide therapeutics is a promising research area for new drug discovery in the pharmaceutical industry. It is attracting increasing interest due to peptide’s high potency, bioavailability, reduced toxicity, drug to drug cross-reactions, and tissue accumulation [26].

Conotoxins are translated from mRNA, and transcriptome sequencing is now the main method for the identification of new conotoxins [27]. Recent studies on the venom duct transcriptome of several *Conus* species have uncovered about 100 conopeptide genes per *Conus* species [28]. In this study, a total of 58 transcripts coding for 73 conopeptides were identified from the transcriptome of *H. tuba*. This is the first report of conopeptide genes in the Buccinidae family, which lay the foundation for further research on characterisation of active compounds for the biological, biotechnological and medical aspect of this species.

Conotoxins are generally classified based on one of three criteria: 1) “gene superfamily”, a classification scheme defined by a highly conserved signal sequence in the protein and evolutionary relationships between conopeptides; 2) “cysteine framework”, a scheme based on the arrangement of cysteines; or 3) “pharmacological family”, this scheme reflects the target specificity of each conopeptide [15].

By definition, conotoxins within a superfamily share a similar signal peptide sequence, but with remarkable structural and functional diversity in the encoded mature peptides [27]. In our study, according to the BlastP results of the signal peptide region, four superfamilies with a strong homology to *Conus* species are reported, the most abundant one being the “Divergent MTFLLLLVSV”. It should be pointed out that, although most of the signal peptide regions were grouped into “Divergent MTFLLLLVSV”, their sequences were distinct from each other, indicating that they may belong to several distinct superfamilies. In addition, conotoxins typically contain a single copy of the mature peptide encoded near the C-terminus [27]. However, several conotoxins found in *H. tuba* exhibit multiple conopeptides (up to three, Figure 4B). The same structure was reported in the disulphide-poor conoCAP (a short peptide with a single disulphide) and numerous other pre-pro-hormone precursor, such as FMRF-amides (H-Phe-Met-Arg-Phe-NH_2_) and enkephalins [27]. The sequence diversity found in *H. tuba* conotoxins may be caused by the genetic divergence to the classical *Conus* species groups, but it could also reflect the existence of a unique superfamily of uncharacterised function (e.g., venom for hunting strategies or as a defensive strategy [29]).

Conotoxins are classified into gene superfamilies by their conserved signal sequence, which is usually associated with a characteristic cysteine framework. Each cysteine framework is in turn associated with a different pharmacological activity [30]. In our study, a total of six patterns of cysteine frameworks were identified, the most abundant one was the type “IX” with six cysteine residues arranged in the pattern “C-C-C-C-C-C”. Although the molecular target of any framework IX conopeptide has not been identified yet, classification research suggests that these peptides are specific to mollusc (some clades of cone snails) and worm-hunting species (turrid and terebrid snails, and some clades of cone snails), but are not produced by fish-hunting cone snail species [31]. *H. tuba* under laboratory conditions appears to be a highly specialised predator feeding on bivalves using different strategies depending on the prey items (e.g., shallow-burrowing or epibyssate species) [10]. The abundancy of the type “IX” cysteine framework of *H. tuba* may be related to the predatory strategies, biotic interactions and evolutionary history of this species. 

The number of disulphide bonds is also one of the important characteristics of conopeptides [26]. It is worth noting that of the 41 unique conopeptides found in *H. tuba*, most (40) were disulphide-rich conopeptides, which contained two or more disulphides; only one disulphide-poor conopressin was identified, where this vasopressin-like peptide may be a good candidate for novel antagonist design because it may act as a selective antagonist to the human V_1a_ receptors [32].

The venom of the Conidae family comprises a complex mixture of hundreds to thousands of conopeptides that are delivered from the venom apparatus for prey capture and self-defence [3]. Surprisingly, in this study, a lot of conotoxin homologs were identified from *H. tuba*, Buccinidae family, which stimulated several interesting questions, for example: What is the evolutionary origin of conopeptide genes? What could be the implications of these genes for *H. tuba*? Does *H. tuba* have the capacity to employ venom in its hunting strategies? If not, what are the potential functions of the conopeptides in *H. tuba* (i.e., are they a defensive strategy)? Also, if they are not venomous in nature, does the discovery of conotoxins in a Buccinidae family member force us to reconsider the current definition of conotoxins? Further investigations are required to understand the underlying molecular mechanisms behind conopeptide origin, functions and structure and predict their targets to ultimately design potential novel conopeptides with specific biotechnological applications [33].

The assembled and annotated transcriptome is a valuable resource for the large-scale discovery of putative functional transcripts and SSR markers for the species [17,22]. In our study, the predicted proteins from the reconstructed transcripts were subjected to BlastP similarity searches against the SwissProt, Pfam, InterPro, KEGG and GO databases. Of the total of 76,306 transcripts, 75,620 (99.1%) were annotated by at least one database, 26,388 (34.6%) were annotated in all five databases, and a total of 42,819 assembled transcripts were allocated into three major GO classes, which provided important candidates for the research of the different functional genes of *H. tuba*. Furthermore, the identification and analysis of SSR markers in the transcriptome will be useful for population genetics to assess the diversity of the resources and help marker-assisted selective breeding, which will have a more immediate impact on species conservation and aquaculture production. In the present study, a total of 20,735 SSR loci were detected in 14,000 unigenes, accounting for approximately 22.7% of the total unigenes. The frequency of transcriptome-derived SSRs in *H. tuba* was higher than in other species of marine mollusc such as *Paphia undulata* (7.5%) [34], *Clithon retropictus* (17.4%) [22] and *Crassadoma gigantea* (19.98%) [35]. Among the potential SSRs, the most abundant type was tri-nucleotide repeats, which was also reported in the Zhikong Scallop (*Chlamys farreri*) and Yesso Scallop (*Patinopecten yessoensis*) [36].

Of the 58 primer pairs designed for microsatellite validation, 23 were polymorphic among 30 individuals of *H. tuba*; this success rate (39.7%) was comparable to previously published transcriptome-derived SSRs in molluscs [35]. The SSRs identified in this study can be valuable for the quantification of genetic diversity within and among wild populations of *H. tuba* and for genetic improvement programs (such as the construction of a genetic linkage map and Quantitative Trait Locus Analysis). SSR markers derived from transcribed regions of DNA are expected to be more conserved and have a higher rate of cross-species applications than genomic SSR markers [37].

In our study, the majority of SSR loci from *H. tuba* revealed cross-species amplification in two other marine gastropods. Thirteen of the 23 markers could be amplified in *H. ternatanus* and 7 in *R. venosa*; the relatively lower cross-species amplification in *R. venosa,* reflect the evolutionary distant with *H. tuba*. Since only one population of each species was used to amplify the microsatellite markers, the polymorphism of these microsatellites might be underestimated. Overall, the SSRs identified in this study will support the study of population resources.

## 4. Materials and Methods

### 4.1. Sample Collection

The visceral mass tissue of an adult female of *H. tuba* collected from Wenzhou, Zhejiang Province, was carefully dissected and immediately placed in liquid nitrogen for RNA preparation. Another 30 wild individuals were sampled from Wenzhou, Zhejiang Province, for SSR polymorphism validation. Furthermore, 16 wild individuals of *Hemifusus ternatanus* collected from Wenzhou, Zhejiang Province, and 20 wild individuals of *Rapana venosa* collected from Qingdao, Shandong Province, were used to test the cross-species amplification. The muscles of these samples were preserved in 100% ethanol until DNA extraction.

### 4.2. RNA Isolation

The frozen tissues were ground into a fine powder in liquid nitrogen and total RNA was isolated using TRIzol reagent (Invitrogen, Waltham, MA, USA) and extracted in accordance with the manufacturer’s protocol. The extracted RNA was treated with RNase-free DNase I (Qiagen, Venlo, Netherlands) to remove the genomic DNA. The purity and concentration of RNA were measured using a NanoDrop-2000 spectrophotometer (Thermo Fisher Scientific, Waltham, MA, USA) and Agilent Bioanalyzer 2100 system (Agilent Technologies, Santa Clara, CA, USA). Approximately 5 µg of RNA was used as the input material for cDNA library construction.

### 4.3. Library Construction and Sequencing

The construction and sequencing of the cDNA library were done by Beijing Genomics Institute (BGI, Beijing, China). Briefly, the poly (A) messenger RNA was isolated from the total RNA samples with oligo (dT) attached magnetic beads (Illumina, San Diego, CA, USA) and the mRNA was fragmented into short fragments using divalent cations under elevated temperature. The cleaved RNA fragments were reverse-transcribed to the first-strand cDNAs by random hexamer primers. Then, the second-strand cDNAs were synthesised to construct the final cDNA library (Illumina, San Diego, CA, USA). After the end repair processing and ligation of the adaptor, RNA was amplified using PCR and purified using QIAquick Gel extraction Kit (Qiagen, Venlo, Netherlands). The cDNA library of visceral mass tissue was sequenced on Illumina Hiseq 2000 platform (Illumina, San Diego, CA, USA) with paired-end reads of 90 nucleotides.

### 4.4. Quality Control and De Novo Assembly

Reads of low quality (i.e., with an average quality score less than 20), having ambiguous bases, being too short, or PCR duplicates were discarded using PrintSeq v0.20.4 [38], and adaptors were clipped using Trimmomatics v0.38 [39]. Ribosomal RNA was further removed using SortMeRNA v3.0.2 [40] against the Silva version 119 rRNA databases [41]. The remaining reads were assembled using Trinity v2.8.4 [42]. The raw assembly was filtered for a minimum transcript length of 300 nucleotides and a detectible CDS with TransDecoder v5.5.0 (https://transdecoder.github.io/). The longest CDS of all the similar alternative splice-form (transcripts) sets was selected as a unigene. Completeness of the assembly was assessed using BUSCO v3 [43] with the Metazoa dataset.

### 4.5. Annotation and Functional Classification

The resulting de novo transcriptome was annotated using InterProscan v5.33-72.0 [44,45], Swiss-Prot release 2018_11 [46] and Pfam release 32.0 database [47]. For classification, the transcripts were handled as queries using Blast+/BlastP v2.8.1 [48], *e*-value threshold of 10^−5^, against Kyoto Encyclopedia of Genes and Genomes (KEGG) release 89.0 [49]. Gene Ontology [50] were recovered from InterPro, KEGG and SwissProt annotations. Subsequently, classification was performed using R v3.5.1 [51].

### 4.6. Conotoxin Identification and Classification

Conopeptides datasets from both ConoServer [52] (6275 peptide sequences, accessed 2019 May 1st http://www.conoserver.org/) and ConoDB (7407 peptide sequences, updated 2018 January 1st; http://conco.ebc.ee) were downloaded and merged. A BlastP v2.8.1 [48] search was conducted using this Conopeptides database (relaxed *e*-value threshold < 10^−5^) and the protein translation of *H. tuba* transcriptome in order to capture the diversity of the conotoxin protein. The putative conopeptide domains were extracted based on the initial BlastP alignment (more than one conopeptide domains been allowed per protein). Each conopeptide was further analysed and kept only if passing a stricter BlastP (*e*-value threshold < 10^−10^), ConoSorter [53] v1.1 or ConoDictor [54]. All conopeptides that passed the filter were aligned using Clustal Omega v1.2.4 [55]. An unrooted phylogenetic tree was inferred using RAxML v8.2.12 [56] under a GTR + I + Γ model of sequence evolution with 10,000 bootstrap replicates. The cysteine (Cys) framework was assigned manually based on Kass et al. [52]. To provide names for conotoxin proteins/genes identified in this study, we used the following naming conventions: two letters to denote the species, the cysteine framework number (or gene superfamily name, when available), and a number denoting the order of discovery within the gene superfamily for that species. Conopeptides followed the same convention, but a lowercase letter was added in the case of multiple domains.

### 4.7. Microsatellite Detection and Validation

To detect perfect simple sequence repeats (SSRs), MISA v1.0 was used (http://pgrc.ipk-gatersleben.de/misa/). SSR loci were identified using the search criteria with the minimum repetitions of di-, tri-, tetra-, penta-, and hexanucleotides being 6, 5, 5, 5, and 5, respectively, and the flanking sequence length of the SSR loci was greater than 100 bp. For SSR validation of polymorphism, primers were designed using the Primer 3 v2.40 [57].

Genomic DNA was isolated from muscles by using the traditional proteinase-K digestion and phenol-chloroform extraction method. The reaction mixture contained 5 μL 2× Power Taq PCR Master Mix (BioTeke, Beijing, China), 100 ng template DNA and 1 μM each primer set in a total volume of 10 µL. PCR amplification was performed with the following program: 5 min at 94 °C; 36 cycles of 1 min at 94 °C, annealing (see Supplementary Table S3 for annealing temperatures) for 1 min, 72 °C for 1 min per cycle and followed by 5 min at 72 °C. The amplified products were separated on 8% denaturing polyacrylamide gel at 1000 V for 4 h and visualised using silver staining. Allele sizes were characterised by using a 10-bp DNA ladder (Invitrogen, Waltham, MA, USA).

The number of alleles, the observed and expected heterozygosity and polymorphism information content (PIC) were calculated by using CERVUS v3.0.3 [58]. Deviations from the Hardy–Weinberg equilibrium (HWE) and linkage disequilibrium (LD) were tested using GENEPOP v4.2 [59]. Sequential Bonferroni corrections [60] were applied for all multiple tests (*p*-value < 0.001).

## 5. Conclusions

This study presents the first assembled and annotated reference transcriptome in *H. tuba*. Multiple conotoxin transcripts were identified and their analysed structure provided potential new sources of bioactive compounds for the pharmaceutical sector. In addition, microsatellite markers were identified and validated in *H. tuba* as well as two other related species (*H. ternatanus* and *R. venosa*). Overall, this study provides the genomic resources and newly discovered conotoxin in *H. tuba* for t future drug development and population resource studies of this species.

## Figures and Tables

**Table 1:** Summary statistics of sequencing and assembly of *H. tuba* transcriptome.

Category	Number/Length
Total number of raw PE reads	33,546,714
Maximum read length (nt)	90
Pre-process PE reads	22,892,498
Cleaned PE reads	21,397,329
Clean bases	1.9 Gb
Transcripts generated (raw)	329,633
Percentage of read assembled	82.9%
Transcripts (filtered)	76,306
Percentage of read assembled	54.5%
GC content	52.9%
Maximum transcripts length	17,498
Minimum transcripts length	300
Transcripts > 500 bp	44,171
Transcripts > 1 kb	17,188
Transcripts > 10 kb	56
N_50_ length (bp)	1014
Mean length (bp)	824.6
Unigenes	61,575
N_50_ length (bp)	865 *
Mean length (bp)	744.2 *

* based on the longest transcript for each unigene.

**Table 2:** Summary of annotation results for *H. tuba* unigenes using a range of databases.

Database	Number annotated
PfamA	60,116
InterPro *	38,711
SwissProt	41,468
KEGG	64,235
GO	42,819
All	26,388
Total	75,620

* InterPro covers 12 databases (CATH-Gene3D, CDD, HAMAP, PANTHER, PIRSF, PRINTS, ProDom, PROSITE (patterns and profiles), SFLD, SMART, SUPERFAMILY, TIGRFAMs).

**Table 3:** Summary of the cysteine framework distribution for the conopeptide and unique conopeptide sequences (details in Supplementary Table S2).

Cysteine Framework	Conopeptide	Unique Conopeptide
*Unclassified*	9	5
NoCys	2	1
I or XXIV	1	1
VIII	7	5
XIV	3	1
XXII	3	1
IX	48	27

**Table 4:** Distribution of the perfect SSR motifs in the *H. tuba* transcriptome.

SSR Type	SSR Number	Unigenes Number	Occurrence (%)	Total (%)
Di-nucleotide	6957	5167	11.3	33.6
Tri-nucleotide	11,654	8418	19.0	56.2
Tetra-nucleotide	1812	1358	3.0	8.7
Penta-nucleotide	278	232	0.5	1.3
Hexa-nucleotide	16	15	<0.1%	0.1
Total	20,735	14,000	33.7	100.0

**Table 5:** Characterisation of successful cross-species amplification of microsatellite loci in two different whelk species, *H. ternatanus* (*n* = 16) and *R. venosa* (*n* = 20). * loci present in the three species.

Species	Locus	Size Range (bp)	N_A_	H_O_	H_E_
*H. ternatanus*	HT4	211-219	4	1.000	0.736
	HT10	209-218	4	1.000	0.690
	HT20	179-189	6	1.000	0.762
	HT22	138-148	6	1.000	0.782
	HT24	212-216	3	0.250	0.232
	HT25 *	168-180	7	1.000	0.867
	HT27	123-137	2	0.563	0.466
	HT28 *	122-128	4	1.000	0.651
	HT29	132-152	10	1.000	0.891
	HT32	249-259	5	0.875	0.718
	HT35	155-159	3	0.688	0.599
	HT36 *	249-261	6	1.000	0.835
	HT39	141-147	4	1.000	0.736
*R. venosa*	HT15	126-136	6	1.000	0.794
	HT23	254-262	5	0.950	0.676
	HT25 *	168-182	8	1.000	0.876
	HT28 *	120-124	3	1.000	0.559
	HT31	117-125	5	1.000	0.788
	HT36 *	245-251	4	1.000	0.740
	HT37	280-290	6	1.000	0.781

N_A_, observed number of alleles; H_O_, observed heterozygosity; H_E_, expected heterozygosity.

## Supplementary Materials

The following are available online at https://www.mdpi.com/1660-3397/17/8/466/s1, Supplementary Table S1: Sequence and structure of the 66 putative conotoxin proteins. Supplementary Table S2: Details of the 82 containing putative conopeptides domains and following characterisation. Supplementary Table S3: Characterisation of 23 polymorphic microsatellite loci in *H. tuba*.
